# An 8-Year-Old Boy With Hepatic Embryonal Sarcoma Managed With Surgical Resection

**DOI:** 10.7759/cureus.68604

**Published:** 2024-09-04

**Authors:** Ali K Alshaya, Rema AlRashed, Ahmad M AlShihri, Helayel Almodhaiberi, Ibrahim Al Hasan, Abdullah A Algarni

**Affiliations:** 1 Department of Surgery, Prince Sultan Military Medical City, Riyadh, SAU

**Keywords:** abdominal mass, malignancy, hepatic, sarcoma, embryonal

## Abstract

The extent of hepatic tumors in childhood differs from that found in older age groups. Some of these tumors are believed to be quite rare like undifferentiated embryonal sarcoma of the liver (UESL). The challenge in diagnosis arises from the nonspecific clinical, biochemical, and radiological features, with definitive diagnosis requiring pathological confirmation following surgical excision. Treatment options with neoadjuvant chemotherapy and surgical resection with satisfactory outcomes have been reported in the literature as well as in our case. We present the case of an 8-year-old boy who initially presented with nonspecific symptoms and was diagnosed with UESL. Following a multidisciplinary team discussion, he was treated with chemotherapy and surgical resection. Post-resection follow-up with MRI revealed stable findings.

## Introduction

Undifferentiated embryonal sarcoma of the liver (UESL) is one of the extremely rare hepatic malignancies, with approximately total of 250 reported cases [1]. It is an aggressive tumor with a peak incidence of 6-10 years of age. It originates from primary mesenchymal tissue [2-3]. It accounts for 2% of hepatic malignancies among all age groups and about 9-13% of hepatic malignant tumors in children [4-5]. The presentation of UESL varies and is not specific. Children mainly complain of abdominal pain or a mass sensation. Some may also present with fever, nausea, anorexia, or loss of weight. [5-7]. The diagnosis of UESL preoperatively in term of clinical, biochemical, and radiological findings is challenging, which mandates a pathological examination of excised or biopsied specimens [3,5]. Surgical management and chemotherapy are the primary options in managing UESL [8]. The disease prognosis is considered to be poor in which early diagnosis and intervention are crucial for better prognosis and survival [3,7]. Therefore, we are presenting a case for an 8-year-old boy, who presented with a fever of unknown origin and abdominal pain which was diagnosed as UESL, in aim to add to literature and aid in diagnosing and managing UESL.

## Case presentation

Our patient is an 8 years old male, not known to have any medical illnesses and he is surgically free. He presented to the emergency department on June 2022 in our hospital with a complaint of fever and abdominal distension. The fever was of a low grade and relieved by antipyretic, associated with malaise, and decreased level of activity. His abdominal distension was progressive over the previous 2 months. There was no history of nausea, vomiting, urinary or bowel motion changes, night sweats, or weight loss. There was no history of recent travel, raw milk ingestion, or contact with animals.

The patient’s family reported that he had been admitted 2 months back as a case of fever of unknown origin, with an impression of infectious underlying process, however all cultures and serology were negative. He has four living siblings, lives with his parents, and his vaccination history is up-to-date. No similar of relevant family history.

Upon examination, the patient was conscious, alert, and oriented to time, place, and person. Vitally normal but febrile, with temperature of 38°C. Not in pain or distress. Not jaundiced. Slightly pale and lethargic. No skin rashes were seen. He had a normal chest examination. Abdominal examination revealed distended abdomen with no scars or engorged veins. There was hepatomegaly reaching to umbilicus, with no abdominal tenderness. No palpable lymph nodes were noticed and bowel sounds were positive. The patient height was 133 cm, weight was 28 kg and BMI of 15.8 kg/m^2^.

**Figure 1:**
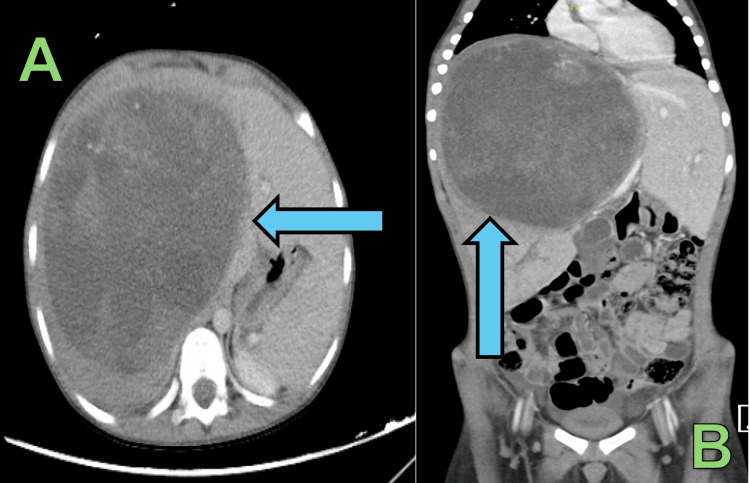
CT abdomen with IV contrast showing hypervascular lesion in the right lobe measuring 15.8 × 12.8 × 16 cm A: Axial view; B: Coronal view

Laboratory investigations were unremarkable except for leukocytosis of 14.6, high ferritin of 402 ng/mL, milady elevated liver enzymes, and high C-Reactive Proteins (CRP) of 133 mg/L (Table 1). An abdomen ultrasound was done and showed enlarged liver measuring 15.3 cm. Moreover, there was a large complex mass lesion containing solid and cystic components extending to the right side of the abdomen without detectable vascularity. The estimated measurement was 17 × 16 × 15 cm, with splenomegaly of 11 cm. He underwent computed tomography (CT) scan of abdomen with a findings of a large complex lesion containing mixed cystic and heterogeneously enhanced necrotic solid components, almost occupying the right liver lobe measuring 15.8 × 12.8 × 16 cm in transverse, anterior-posterior (AP), and craniocaudal dimensions respectively, causing mass effect on liver parenchyma, porta hepatis and on the right kidney with no calcification (Figure 1). The mass showed prominent arterial supply from the right and left hepatic arteries, with enhancement of the solid component and septations. The intrahepatic inferior vena cava (IVC) was compressed and deviated medially. The mid and right hepatic veins were not visualized which were likely compressed. The right portal vein appeared significantly compressed but showed contrast opacification. The left portal vein and the left hepatic vein were spared. However, they were mildly compressed. Intrahepatic duct dilatation was noted in the right and left hepatic lobes. No size significance abdominopelvic lymphadenopathy, and no other organomegaly nor other masses.

**Table 1:** Laboratory investigation

Item	Value	Item	Value
White blood counts (WBCs)	14,600/mm^3^ (Neutrophils 82.9)%	Alanine transferase (ALT)	64 U/L (0-40)
Hemoglobin	10.7 g/L (11.5-15.5)	Alkaline phosphatase (ALP)	126 U/L (142-335)
Platelets	825,000/mm^3^ (150-450)	Total bilirubin	17 mcmol/L(2-5)
Albumin	39 g/L (38-54)	Direct bilirubin	11 mcmol/L (1-5)
International normalized ratio	1.7 (0.9-1.3)	Lactate dehydrogenase (LDH)	467 U/L (140-280)
Ferritin	402 ng/mL (12-150)	C-reactive protein) (CRP)	133 mg/L (8-10)
Alpha fetoprotein	0.8 ng/mL		

CT chest done and showed linear atelectatic and mosaic ground-glass attenuation of the lungs, which could be a sequela of small airway disease/bronchiolitis, no gross pulmonary nodules or masses could be seen.

**Figure 2:**
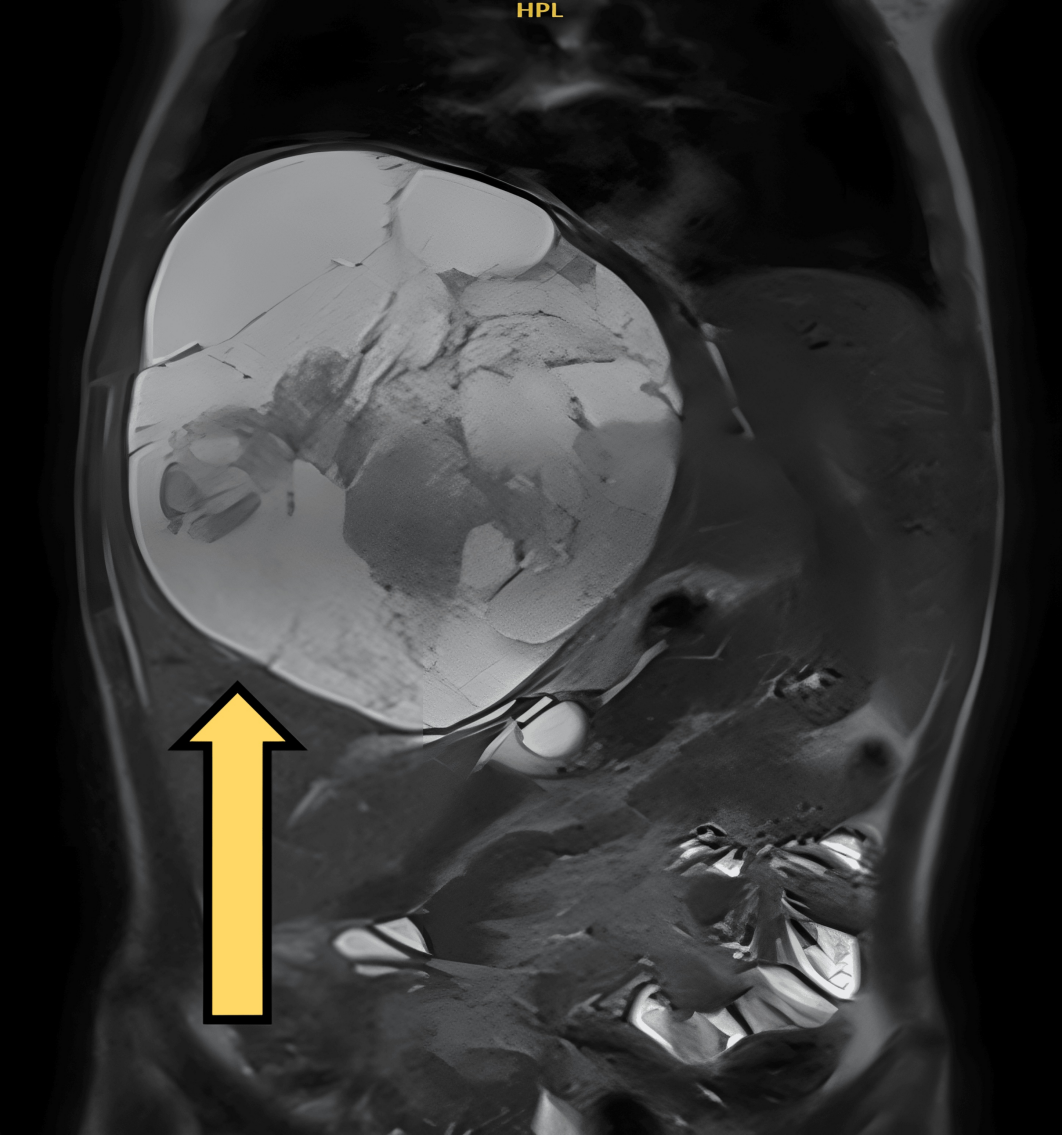
Coronal view of MRI abdomen showing well-defined encapsulated mass of mixed signal intensities measuring approximately 17.6 × 14.3 × 15 cm

Then MRI of the abdomen was done (Figure 2) and it showed a huge well-defined encapsulated mass of mixed signal intensities and patchy enhancement occupying the right hepatic lobe with relative preservation of segment 6 resulting in elevation of the right hemidiaphragm. The left hepatic lobe is preserved. This mass measured approximately 17.6 × 14.3 × 15 cm at craniocaudal, mediolateral, and anteroposterior diameters respectively, with an estimated volume of 1.888 cc. It was causing mass effect on the adjacent structures. Central intrahepatic biliary dilatation was noted and the gallbladder was markedly distended with surrounding pericholecystic fluid.

The impression was of aggressive neoplastic liver mass, likely representing undifferentiated embryonal sarcoma, other differential diagnoses include hepatoblastoma, hepatocellular carcinoma which is less likely without underlying chronic liver disease. Hepatic mesenchymal hamartoma was considered as a differential diagnosis too. The appearance of the mass was not typical for a hydatid cyst or liver abscess.

A surgical consultation was done and after reviewing the images, resection of this mass was not feasible due to size of the lesion and effect on the right hepatic vein. After discussion in a multidisciplinary meeting, the decision was to obtain and histological diagnosis. Liver biopsy under ultrasound guidance was done and a pathological diagnosis of UESL was made. Tumor board was conducted again to discuss the case as non-metastatic UESL and decision was to go for neoadjuvant chemotherapy prior to hepatic resection Vs. liver transplantation. Patient was started on neoadjuvant chemotherapy (IFOSFAMIDE/DOXORUBICIN) for a total of 10 weeks, 4 cycles.

**Figure 3:**
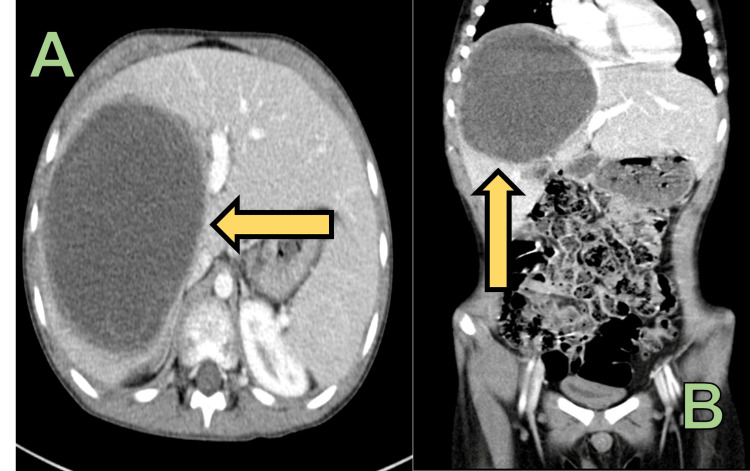
CT abdomen with IV contrast showing significant interval decrease in the size of the right hepatic lobe mass to 11.4 × 10.8 × 12.6 cm A: Axial view; B: Coronal view

On follow-up imaging, there was a significant interval decrease in the size of the right hepatic lobe mass in segment VIII, VII and V, to 11.4 × 10.8 × 12.6 cm compared to 15.8 × 12.8 × 16 cm in AP, transverse and craniocaudal dimensions respectively. The calculated volume is 722 cc compared to the previous volume of 1888 cc. The compression of left portal vein and left hepatic vein have significantly improved compared to pre-chemo imaging (Figure 3).

A surgical re-evaluation was done and after reviewing the reduction of the mass to almost 50% of size in CT scan, the patient was planned for resection. Patient underwent exploratory laparotomy and extended right hepatectomy was done on 09/11/2022. Estimated intra-operative blood loss was 300 cc, and operative time was 5 hours.

Postoperatively, the patient was shifted to Pediatric Intensive Care Unit (PICU) for 24 hours for observation then he was shifted back to the ward. He developed atelectasis of the right lung with high respiratory rate, however he was managed with chest physiotherapy and pain killer and completely resolved. He was discharged on 21/11/2022 with hepatobiliary surgery clinic follow-up.

Later on, his pathology report showed a few residual viable tumor cells representing less than 1% of the whole mass in extensive fibrous scarring. Tumor was confined to the liver. No vascular invasion is identified. The liver capsule is intact. Resection margin is negative for a viable tumor (1.3 cm away). Therapy-related changes including tumor necrosis, hyalinization, fibrous spheres, microcalcification, hemosiderin-laden macrophages, and foamy histiocytes are seen. Adjacent hepatic parenchyma with changes secondary to mass effect. Portal vein lymph node was reactive lymph node with NO evidence of malignancy (0/1).

He was then referred to oncologist where he received adjuvant chemotherapy (IFOSFAMIDE), for a total of two cycles, last cycle was completed in January 2023.

Patient is planned to be followed with an MRI abdomen every 3 months in the first 2 years and then every 6 months and then every year. Last follow-up MRI abdomen was done on Jan 2024 and it showed no recurrence with stable splenomegaly.

## Discussion

UESL arises from primary mesenchymal tissue. It was first described by Stocker and Ishakand in 1978. It is considered to be one of the rarest and one of the most aggressive malignant hepatic tumors [1-3]. In pediatrics, UESL accounts for 2% of liver malignancies with a lower incidence in adults [4,9]. It is found to be more common in the right lobe of the liver, as it is in our case [10]. UESL has nonspecific symptoms as we described in our case [11,12]. However, it was reported in the literature that it can present more dramatically and acutely with mass rupture and intra-abdominal hemorrhage requiring an urgent intervention [13-15]. Making the diagnosis more challenging, there is no specific tumor markers known for UESL. On the other hand, it might have laboratory readings of high LDH as it was in our patient (LDH was 467 U/L). Some studies showed that AFP might be elevated which was not consistent with our case [16-18].

Imaging modalities are required for the diagnosis of UESL. In our case the CT abdomen showed a large complex lesion containing mixed cystic and heterogeneously enhanced necrotic solid components, which might be confusing with other diagnoses like hydatid cyst, hamartoma or biliary cystadenoma. This along with nonspecific clinical presentation can lead to delay or misdiagnosis which in turn can affect the overall prognosis and survival [4-7,10]. The delay in diagnosing UESL can reach up to 23.5% [19-22].

Histopathological examination of an obtained liver sample is essential in diagnosing UESL. Under microscope, as oval irregular cells with multinucleated cells with presence of acid Schiff (PAS) positive diastase-resistant hyaline globules is helpful in diagnosis, which was consistent with our pathological reports [23].

In immunohistochemistry, there are no specific stains that can help in distinguishing UESL from other tumors, unfortunately it was not done to our specimen [6,24,25].

A common differential diagnosis that should be excluded especially in pediatric age groups include hepatoblastoma and hepatocellular carcinoma. Immunohistochemistry staining is not specific for UESL. however, it should be included in the workup to roll out other possible hepatic lesions [10,11].

Surgical management is the mainstay of the treatment of UESL. Chemotherapy nowadays is commonly used alongside surgical resection with better outcomes in patients diagnosed with UESL [7,9]. Neoadjuvant chemotherapy and radiation have increased the survival up to 70-100% [26]. Ifosfamide and doxorubicin is widely used for the neoadjuvant chemotherapy for soft tissue sarcomas as in our case [27]. Moreover, radiotherapy can be used in case of metastasis or to prevent the recurrence of the tumor, but its effectiveness is not well studied. For those patients who are found to have unresectable disease, liver transplantation is considered as a possible option [7,9].

## Conclusions

UESL is considered to be an aggressive and rare tumor. It is arising from mesenchymal hepatic tissue. It has nonspecific clinical, radiological and pathological features, which can delay the diagnosis and make it challenging. Pathological examination of a sample obtained from liver is required to diagnose UESL. Hepatoblastoma and hepatocellular carcinoma are commonly considered as differential diagnosis. Surgical management as well as chemotherapy are the mainstay of treatment.
